# Response of coagulation and fibrinolysis system was different between older and nonolder patients with severe sepsis

**DOI:** 10.1186/cc13293

**Published:** 2014-03-17

**Authors:** D Kudo, S Yamanouchi, T Sato, R Nomura, T Omura, N Miyagawa, S Kushimoto

**Affiliations:** 1 Tohoku University Hospital, Sendai, Japan

## Introduction

The present study investigated the relationship between age [1] and biomarkers of coagulation and fibrinolysis [2] and their impact on outcome in severe sepsis patients.

## Methods

A prospective observational study of adult patients with severe sepsis was conducted in a single academic hospital. Plasma was analyzed for coagulation and fibrinolysis markers on days 1, 2, and 3. Patients were stratified according to the age 67 years, which was the point of the Youden index (maximum sensitivity + specificity - 1) in a receiver operation characteristics plot for a logistic regression model of in-hospital mortality.

## Results

For the in-hospital survival rate, that of older sepsis patients was significantly lower than younger patients, 9/15 (60.0%) versus 27/28 (96.4%), *P *< 0.05. Older patients had markedly higher total plasmin activator inhibitor-1 (TPAI-1) on day 1, thrombin-antithrombin complex (TAT) on days 2 and 3, and fibrin monomer complex on day 2, and markedly lower plasminogen (PMG) on day 3 and alpha-plasmin inhibitor (aPI) on days 2 and 3 compared with younger patients (all *P *< 0.05). Age was an independent predictor of high TAT on days 2 and 3, high fibrin monomer complex on day 2, low PMG on day 3, and low aPI on days 2 and 3 after adjusting for some cofactors and covariables. TPAI-1 on day 2, TAT on day 2, and PMG on day 3 were risk factors of inhospital mortality in older sepsis patients (Table 1).

**Table 1:** Univariate analysis of logistic regression for in-hospital mortality

	HR	95% CI	***P***
PAI-1 day 2	1.02	1.0 to 1.06	<0.01
TAT day 2	1.49	1.02 to 19	<0.05
PMG day 3	0.64	0.1 to 0.95	<0.01
SOFA day 1	1.2	0.9 to 1.69	0.21
IL-6 day 1	0.99	0.9 to 1.00	0.21

## Conclusion

In severe sepsis, older patients displayed a biomarker profile suggestive of enhanced response of coagulation and fibrinolysis system compared with younger patients. Change of some markers depended on age and may contribute to the poor outcome in older patients.
